# Dental Students’ Perceptions of Workforce Readiness, Career Aspirations and Institutional Support Needs at the Point of Professional Transition: A Cross-Sectional Study in Romania

**DOI:** 10.3390/dj14050300

**Published:** 2026-05-14

**Authors:** Băluță Daniel, Dragomirescu Anca Oana, Drăgoi Mihaela Cristina, Băluță Andreea Mihaela, Păcurar Mariana, Ionescu Ecaterina

**Affiliations:** 1 Department of Restorative Odontotherapy, Faculty of Dental Medicine, “Carol Davila” University of Medicine and Pharmacy, 020021 Bucharest, Romania; 2 Department of Orthodontics and Dentofacial Orthopaedics, Faculty of Dental Medicine, “Carol Davila” University of Medicine and Pharmacy, 020021 Bucharest, Romania; 3 Department of International Business and Economics, Faculty of International Business and Economics, Bucharest University of Economic Studies, 010404 Bucharest, Romania; cristina.dragoi@rei.ase.ro; 4 Department of Esthetic Dentistry, Faculty of Dental Medicine, “Carol Davila” University of Medicine and Pharmacy, 020021 Bucharest, Romania; 5 Department of Orthodontics, Faculty of Dental Medicine, George Emil Palade University Medicine, Pharmacy, Science, and Technology of Târgu Mureș, 540139 Târgu Mureș, Romania; mariana.pacurar@umfst.ro

**Keywords:** dental students, professional transition, workforce integration, institutional support, public authorities, career choice, migration intentions

## Abstract

**Background**: The transition from dental education to professional practice represents a critical stage in career development, influenced by individual expectations, labor market conditions, and institutional support mechanisms. This study aimed to explore final-year dental students’ perceptions of professional transition and the role of public authorities in facilitating early-career integration. **Methods**: A cross-sectional study was conducted among 216 final-year dental students from a single Romanian university using a structured, self-administered questionnaire. Descriptive and inferential statistical analyses were performed using Jamovi software, with significance set at *p* < 0.05. **Results**: Most students reported feeling insufficiently prepared for professional practice and identified lack of clinical experience as the main barrier to employment. A strong preference for private sector employment was observed, while interest in the public sector was limited. Students expressed a clear need for structured support, including mentorship, practical training, and career guidance. A significant association was identified between intention to work abroad and the types of support expected from authorities (χ^2^(2) = 14.7, *p* < 0.001, moderate effect size). **Conclusions**: The findings highlight important challenges in the transition to professional practice and emphasize the need for coordinated interventions involving educational institutions and public authorities. Strengthening structured support mechanisms may facilitate professional integration and contribute to improved workforce retention.

## 1. Introduction

The transition from university education to labor market integration represents a critical stage in professional development within the healthcare sector. Evidence from human resources for health research indicates that early career stages are often characterized by uncertainty, challenges in employment integration, and a need for institutional support, constituting a key bottleneck in retaining young professionals [1,2,3,4,5]. In dentistry, this transition is particularly complex, requiring not only clinical competencies but also practical, entrepreneurial, and practice management skills [2,6].

In the context of ongoing transformations in healthcare systems and increased professional mobility within the European Union, interest in the career aspirations of medical students has grown. International studies show that career choices in dentistry are influenced by factors such as professional development opportunities, working conditions, remuneration, and work–life balance [2,3,6,7,8]. At the same time, graduates frequently perceive a gap between academic training and labor market demands, particularly regarding practical and entrepreneurial competencies [6,9,10,11].

Professional migration represents a relevant phenomenon in Central and Eastern Europe, including Romania, where decisions to work abroad are shaped by both economic considerations and perceptions of professional opportunities and institutional support [8,12,13]. Studies among Romanian medical students indicate a significant intention to migrate, often linked to perceived limitations in local career opportunities [14]. Structured professional support mechanisms—such as mentorship, postgraduate training, and structured transition programs—have been associated with improved professional adaptation and job satisfaction [2,4,15].

In Romania, research on the professional integration of dentists remains limited, with existing studies focusing mainly on migration and workforce distribution. Data on students’ perceptions of labor market integration and the role of public authorities in this process are still scarce, despite their importance for informing workforce policies and reducing territorial disparities in access to care [4]. In this context, recent investments in modern educational and clinical facilities for dental students at the Faculty of Dentistry of the Carol Davila University of Medicine and Pharmacy (UMFCD), Romania, have created an opportunity to explore how support frameworks are perceived by final-year students, who represent the first beneficiaries of these developments.

Therefore, the aim of this study was to explore the perceptions of final-year dental students regarding their professional future, focusing on career aspirations, perceived preparedness, job selection criteria, and expectations concerning institutional support, particularly the role of local public authorities in facilitating the transition to professional practice.

## 2. Materials and Methods

The study sample consisted of 216 final-year students (fifth and sixth year) from the Faculty of Dentistry at UMFCD. Student participation was voluntary, and responses were anonymous, being used solely for academic and research purposes. This study focuses exclusively on Romanian students enrolled in the Romanian-language program. This choice is justified by the fact that Romanian students have a distinct institutional and social relationship with local public authorities compared to foreign students, and the study specifically aims to assess perceptions related to this context.

The anonymous questionnaire included 12 questions concerning targeted professional directions, job selection criteria, migration intentions, and perceptions regarding the role of public authorities in facilitating the professional integration of young dental professionals. The questionnaire was developed based on a review of relevant literature and underwent a pretesting phase to evaluate clarity and face validity. Feedback obtained during this phase was used to refine item wording and structure before final deployment. Sample size was calculated using the Cochran standard formula for finite populations, assuming a 95% confidence level, a 5% margin of error, and a response distribution of 50%. The minimum required sample size was estimated at 214 participants. To account for potential non-response or incomplete questionnaires, a higher number of participants was targeted.

Data collection was conducted using an online questionnaire created on the Google Forms platform and distributed via institutional email to fifth- and sixth-year students at the Faculty of Dentistry, UMFCD. The study was approved by the Ethics Committee of the Carol Davila University of Medicine and Pharmacy, Bucharest (approval no. 16351/1 July 2025). Participation was voluntary, and informed consent was obtained from all participants. Statistical processing was performed using Jamovi software (version 2.x). The analysis included descriptive statistics (absolute and relative frequencies, graphical representations) for all investigated variables, as well as inferential analyses based on the Chi-square test of independence and Fisher’s exact test, used to assess associations between categorical variables. The assumptions for the Chi-square test were verified prior to analysis. Effect sizes (Cramér’s V) were calculated for significant associations to assess the strength of relationships. Several questionnaire items (Q4, Q6, Q9, Q11, and Q12) allowed multiple responses, and results were analyzed accordingly. Due to the large number of potential variable combinations, inferential analyses were limited to selected conceptually relevant associations in order to reduce the risk of type I error and improve interpretability. Statistical significance was set at *p* < 0.05.

## 3. Results

This study is part of a broader doctoral research project. Beyond the variables included in the questionnaire, the research analyzed numerous correlations that help outline an accurate profile of future dentists at University of Medicine and Pharmacy “Carol Davila” (UMFCD) regarding their professional aspirations. Due to space limitations, this article presents a synthesis of results deemed relevant for drawing meaningful conclusions and suggesting corresponding measures.

**Figure 1:**
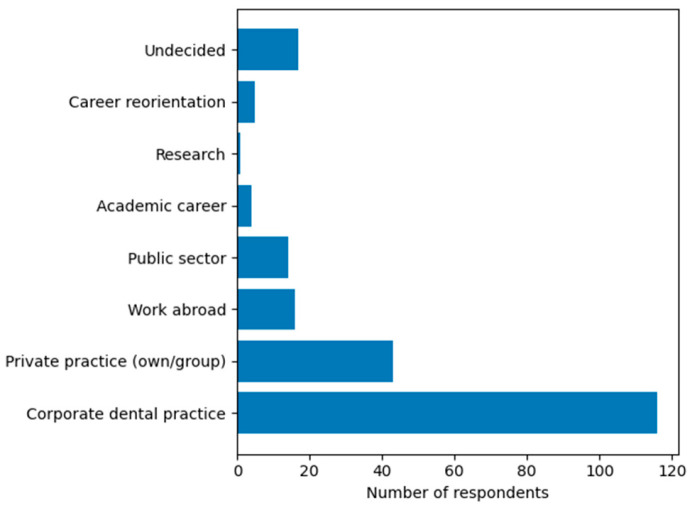
Preferred professional direction after graduation.

**Figure 2:**
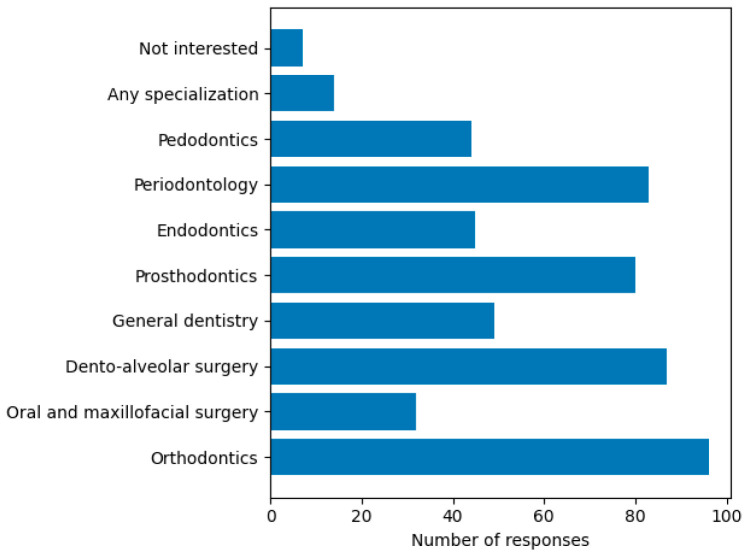
Preferred dental specializations among respondents (multiple responses allowed).

Participant Characteristics (Q1). A total of 216 final-year dental students participated in the study, resulting in a response rate of 45%. The majority of respondents were female (77.8%, n = 168), followed by male students (21.3%, n = 46), while 0.9% (n = 2) preferred not to disclose their gender.
Distribution by academic year (Q2). Distribution by academic year showed that 34.3% of respondents were fifth-year students (n = 74), while the majority of participants were in their sixth year (65.7%, n = 142).
Preferred Professional Direction (Q3). Most respondents preferred employment in the private sector, particularly within corporate dental practices (53.7%), followed by private practice (19.9%), while other options were selected by smaller proportions of students (Figure 1).
Preferred Dental Specializations (Q4)**.** Regarding preferred dental specializations (Figure 2), respondents indicated interest in multiple clinical fields, reflecting diverse professional aspirations. Orthodontics was the most frequently selected specialization, followed by dento-alveolar surgery, periodontology, and prosthodontics (Figure 2).

Perceived Preparedness for Labor Market Entry (Q5). Regarding perceived preparedness for entering the labor market, 46.3% of respondents reported feeling unprepared, while 34.3% felt only partially prepared. A smaller proportion of students considered themselves fully prepared.
Job Selection Criteria (Q6). The most important criteria for job selection were salary and professional development opportunities (both 75.5%), followed by a supportive work environment and work–life balance (Table 1).
Preferred work environment: rural vs. urban (Q7). With regard to the urban–rural variable, it is noteworthy that more than three quarters of students indicated a preference for working in an urban environment (75.9%, n = 164), while only four respondents expressed a preference for working in rural areas. For 22.2% of students (n = 48), the location of their future medical practice—urban or rural—was not considered an important factor.
Intention to work abroad (Q8). Regarding the intention to work abroad after graduation, the analysis indicates that only 14.8% of respondents (n = 32) expressed a clear intention to work abroad. In contrast, 37% (n = 80) of future graduates stated that they do not intend to work outside the country, while nearly half of the respondents (48.1%, n = 104) indicated that they do not exclude the possibility of working abroad after graduation.

**Table 1:** Job selection criteria (multiple response).

Criterion	n	%
Salary/Income	163	75.5
Professional development opportunities	163	75.5
Friendly work environment	161	74.5
Respect and appreciation of the profession	157	72.7
Work–life balance	151	69.9
Flexible schedule	139	64.4
Job security	135	62.5
Opportunity to apply acquired skills	126	58.3
Geographic location	117	54.2
Private sector	78	36.1
Personal fulfillment/attachment to patients	71	32.9
Clinic reputation	51	23.6
Public sector	19	8.8
Other (e.g., financial motivation statements)	2	1

**Figure 3:**
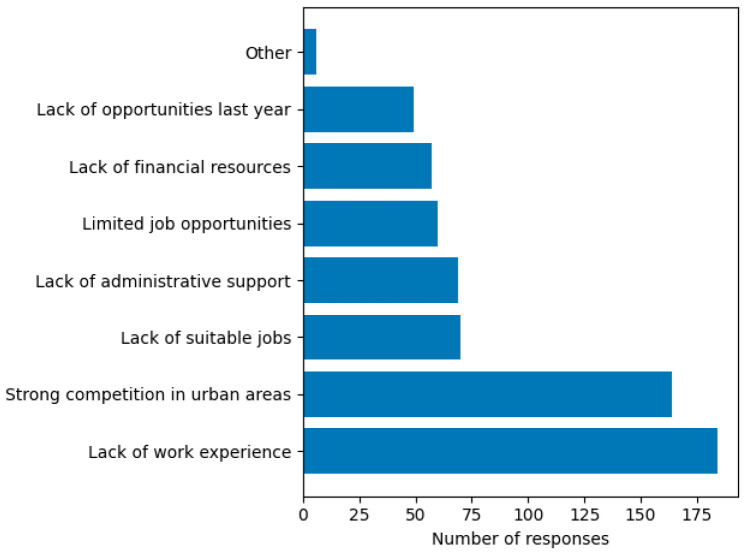
Difficulties identified in choosing a workplace after graduation (multiple responses allowed). Minor responses (n = 1 each) were grouped under “Other”.

Difficulties in choosing a workplace after graduation (Q9). The descriptive analysis of perceived difficulties in choosing a workplace after graduation (Figure 3) indicates that the lack of professional experience at the time of employment represents the main challenge identified by students, being mentioned by 85.2% of respondents (n = 184).

**Figure 4:**
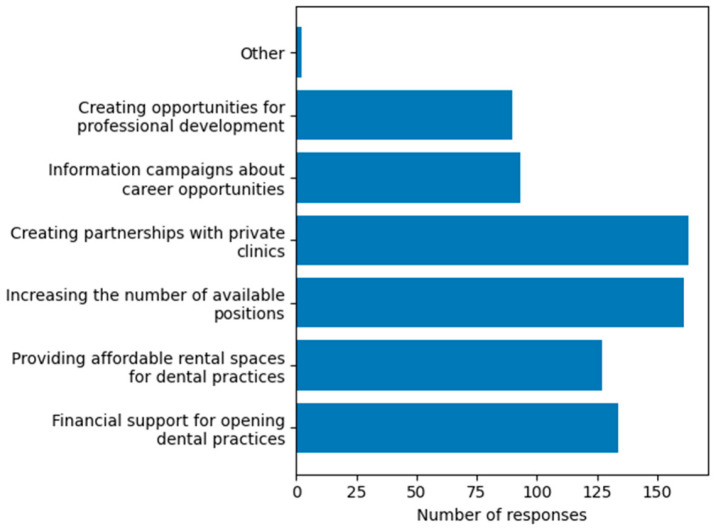
Expected forms of support from local public authorities (multiple responses allowed). Minor responses (n = 1 each) were grouped under “Other”.

Perceptions of institutional effectiveness (Q10). The analysis of responses regarding the involvement of local authorities in facilitating the professional integration of graduates, another variable investigated in this study, reveals a clear trend. The majority of students (83.3%, n = 180) consider the involvement of local public authorities necessary in supporting their transition to the labour market. In contrast, only a very small proportion of respondents (3.24%, n = 7) believe that such involvement is not necessary, while 13.4% (n = 29) reported that they do not have a formed opinion on this issue.
Expected forms of support from local public authorities to facilitate labour market integration of young dentists (Q11). The most frequently selected forms of support included partnerships with private clinics (75.5%) and increasing the number of available positions (74.5%) (Figure 4).

**Figure 5:**
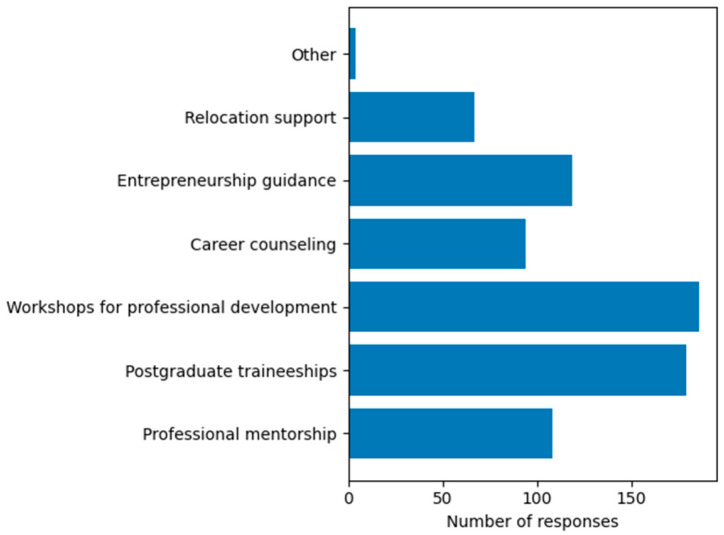
Types of support expected for professional development (multiple responses allowed).

Types of institutional support considered useful for professional development (Q12). The most frequently selected forms of support were workshops for professional development (86.1%) and postgraduate traineeships (82.9%) (Figure 5).

Inferential analyses were performed to examine selected associations between students’ professional orientation and their expectations regarding institutional support. Given the exploratory nature of the study, only conceptually relevant relationships were tested, in order to reduce the risk of type I error and improve interpretability.

Associations between job selection criteria (Q6) and expected forms of institutional support (Q12) are summarized in Table 2. Several statistically significant associations were identified. The importance of salary was associated with career counseling and entrepreneurship guidance. Geographical location was associated with career counseling and entrepreneurship support, while preference for the private sector was strongly associated with entrepreneurship support (χ^2^(1) = 16.0, *p* < 0.001, moderate effect size) and mentoring. No significant associations were observed for other variable combinations (all *p* > 0.05).

Associations between preferred professional direction (Q3) and expected forms of institutional support (Q12) were analyzed using Chi-square tests (Table 3). Significant associations were identified. Preference for employment in the private sector (Q3) was associated with interest in entrepreneurship support (Q12) (χ^2^(1) = 16.0, *p* < 0.001) and mentoring programs (χ^2^(1) = 5.14, *p* = 0.023, small effect size). Students expressing interest in the public sector (Q3) were more likely to indicate a need for relocation support (Q12) (χ^2^(1) = 7.34, *p* = 0.007, small effect size). No statistically significant associations were observed between other professional directions and forms of structured professional support (all *p* > 0.05).

The association between intention to work abroad (Q8) and expected support (Q12) was analyzed using Chi-square tests (Table 4). Inferential analysis revealed a statistically significant association between students’ intention to work abroad (Q8) and the types of support expected from public authorities (Q12) (χ^2^(2) = 14.7, *p* < 0.001, moderate effect size). No statistically significant associations were identified between Q8 and other forms of support frameworks (all *p* > 0.05).

## 4. Discussion

The present study explored the perceptions of final-year dental students regarding their professional transition and integration into the labor market, with a particular focus on career expectations, perceived preparedness, and the role of institutional support, including local public authorities. While the literature includes numerous studies on dental students’ career aspirations and workforce readiness, the role of public and local authorities in facilitating this transition remains insufficiently explored, particularly in the Romanian context.

### 4.1. Professional Orientation and Labor Market Structure

One of the most prominent findings is the strong preference for employment in the private sector, particularly within corporate dental practices. This pattern may reflect the current organization of dental services in Romania, which are largely perceived by students as being delivered through private providers—corporate networks, which are considered to offer professional stability, operational infrastructure, and rapid integration into the labor market—a phenomenon also described in the literature regarding the integration of young clinicians into large organizations [7], but which may also lead to a profile oriented toward immediate professional stability, with avoidance of the risks associated with initiating one’s own practice [7,16,17,18]. However, the results of our study differ from those reported in the study by Halawany (2017), with the preference for private practice being influenced by the presence of a dentist within the family [19]. These differences may also reflect cultural and social particularities between Europe and the Arab world [19]. Similar trends have been reported in countries such as Germany and the United Kingdom, where young dental professionals tend to favor work environments offering financial stability, access to infrastructure, and structured career development pathways [2,6,7]. At the same time, the relatively low proportion of students considering employment in the public sector suggests not necessarily a lack of interest, but rather a limited perceived attractiveness and visibility of this sector. This may be associated with structural factors such as limited employment opportunities, lower remuneration, or reduced access to modern clinical infrastructure. These findings highlight the need to reconsider the positioning of the public dental sector as a viable career pathway for young professionals.

### 4.2. Perceived Preparedness and Transition Gap

A substantial proportion of respondents reported feeling unprepared or only partially prepared for entering the labor market. This finding is consistent with previous studies documenting a gap between academic training and the practical demands of clinical practice [6,9,10], and reinforces the challenges associated with the transition from academic training to professional practice, as highlighted in previous research. In dental education, this gap is often associated with limited exposure to real-life clinical scenarios, insufficient opportunities for independent practice, and inadequate training in practice management and entrepreneurship. The strong demand identified in this study for practical workshops, postgraduate traineeships, and structured transition programs reinforces the existence of this gap. Similar findings have been reported internationally, where transition support mechanisms—such as mentorship programs and supervised early-career practice—are associated with improved confidence, better professional adaptation, and increased job satisfaction among young healthcare professionals [2,4,15].

### 4.3. Job Selection Criteria and Early-Career Priorities

The analysis of job selection criteria highlights a pragmatic orientation among students, with salary and professional development opportunities being the most important factors. The distribution of preferred dental specializations also suggests a tendency toward clinically and professionally attractive fields, which may reflect perceived labor market opportunities and expectations regarding career development. These findings align with international research indicating that financial considerations, career progression, and work–life balance play a central role in early-career decision-making in healthcare professions [5,6,20]. In the study by Crossley and Mubarik (2002), dental students demonstrated a stronger orientation toward personal and financial gain compared to medical students, who exhibited a more professional attitude in which altruism and intellectual challenge constituted central motivational factors [21]. In contrast, in another study by Shaikh in 2018, the motivation behind choosing a career in dentistry was the desire to help people regardless of other considerations, while financial aspects were ranked last [22]. The results of our study are consistent with those reported by Batyrbekova et al. (2022), particularly regarding students’ predominant orientation toward achieving a balance between professional and social life and ensuring economic stability [23]. Importantly, the inferential analysis suggests that students who prioritize financial outcomes are more likely to seek structured career guidance and entrepreneurial support, indicating a more strategic approach to career planning. This finding supports the need for targeted interventions that address not only clinical competencies but also financial literacy, business skills, and long-term career planning.

### 4.4. Migration Intentions and Professional Uncertainty

Although only a minority of students expressed a definite intention to work abroad, a substantial proportion indicated uncertainty regarding this decision. This pattern suggests that migration is not a fixed choice, but rather a potential option influenced by evolving perceptions of professional opportunities and working conditions. This “potential mobility” is a well-documented phenomenon in Central and Eastern Europe, where migration decisions are often not fixed but influenced by evolving perceptions of professional opportunities, working conditions, and institutional support [13,14,24]. This pattern is consistent with previous findings reported in Romania and other Central and Eastern European countries [14]. There are also several studies conducted in Romania or focusing on Romania that analyze physician migration (“brain drain”), most of which conclude that the phenomenon is associated with income levels and conditions within the healthcare system; however, increased healthcare expenditure and higher salaries may reduce migration [24,25,26]. From the perspective of the present study, this interpretation is supported by the association identified between the intention to work abroad (Q8) and the need for professional mentorship (Q12). The relationship between perceived barriers and migration intentions suggests that addressing early-career challenges—particularly lack of experience and competition in urban areas—may play a role in reducing the intention to seek employment abroad. In this context, the presence of a large proportion of undecided respondents may represent an important window of opportunity for targeted interventions aimed at improving early-career conditions and professional integration. Strengthening institutional support mechanisms may contribute not only to individual career development but also to improving workforce retention at the national level.

### 4.5. Barriers to Labor Market Integration

The predominance of lack of professional experience as the main perceived barrier highlights the central challenge of transitioning from student to independent practitioner. This finding is consistent with previous studies showing that newly graduated dentists often face difficulties in gaining clinical autonomy and securing stable employment [1,6,23]. Additionally, the perception of high competition in urban areas reflects structural imbalances in workforce distribution. While most graduates prefer urban practice, this concentration may contribute to oversaturation in certain regions and insufficient coverage in others. The strong preference for urban environments, combined with the limited attractiveness of rural areas among future graduates, represents a critical issue with potential implications for the future distribution of healthcare personnel. This aspect raises concerns for public authorities, given that a significant proportion of the population resides in rural areas, similar findings being also reported in other studies [27,28,29,30,31]. These results underline the importance of policy interventions aimed at improving workforce distribution and incentivizing practice in underserved areas. This finding is closely related to the perceived lack of preparedness reported by students, reinforcing the existence of a mismatch between training and professional demands between academic training and professional practice. At the same time, these barriers may contribute to the uncertainty observed in migration intentions, suggesting that difficulties encountered at the beginning of the professional career may influence decisions regarding employment both within the country and abroad.

### 4.6. Institutional Support and the Role of Public Authorities

A key contribution of this study is the identification of a strong expectation among students regarding support frameworks, particularly from local public authorities. This is further supported by the observation that most respondents perceived the involvement of local authorities as necessary for facilitating their transition to professional practice, indicating a gap between educational outcomes and available support mechanisms for professional integration. Overall, these results support the growing body of evidence highlighting the importance of institutional support mechanisms in facilitating professional integration among young healthcare professionals. Students identified several relevant forms of support, including partnerships between universities and dental practices, increased opportunities within the public sector, and support for establishing independent practices, reflecting their expectations regarding how local authorities could facilitate professional integration. These expectations are consistent with international evidence suggesting that local and regional policies can play a significant role in facilitating workforce integration and improving access to healthcare services [4,32,33]. In this context, the World Health Organization launched a program in 2025 promoting local strategies for strengthening the health workforce, involving local authorities, universities, and medical institutions in workforce planning and retention [34]. Importantly, the types of support preferred by students are predominantly practice-oriented, such as mentorship, postgraduate training, and practical workshops. These preferences reflect the types of support for individual professional development expected from authorities, as reported by participants in the present study. This suggests that students do not primarily expect administrative interventions, but rather structured support mechanisms that directly facilitate their transition into professional practice. The limited interest in the public sector represents a relevant finding with potential implications for healthcare policy. This aspect may warrant increased attention from public institutions responsible for education and healthcare planning. Expanding the availability and visibility of public dental services could contribute to creating viable career alternatives, while also improving access to care, particularly for disadvantaged populations. The context of recent investments in modern educational and clinical facilities at UMFCD may also be relevant for interpreting these findings, as improved training environments could influence students’ expectations regarding institutional support and professional integration. A recent initiative at the local level illustrates this direction, through the establishment of a large outpatient public dental clinic in Bucharest, developed by local authorities and operational since 2024. Although this model is still recent, it provides a relevant framework for future evaluation of whether increased public-sector opportunities may influence the professional orientation of dental graduates over time. These considerations are consistent with previous research suggesting that physicians attracted to the public sector tend to prioritize professional stability and the social role of the profession, while those oriented toward the private sector value autonomy and economic opportunities [14].

### 4.7. Gender Distribution and Implications

The predominance of female respondents (77.8%) in this study reflects a broader trend observed in dental education, where female students are increasingly overrepresented [14,35,36,37]. This phenomenon has been reported in multiple European countries, with their proportion frequently exceeding 60–70%, and is associated with long-term shifts in workforce composition and professional dynamics. Moreover, the specialized literature highlights a global trend toward the feminization of the profession, with women currently representing approximately 48–75% of the dental workforce worldwide, this proportion varying depending on the national context [38]. In some European countries, this trend is even more pronounced (in Finland, over 75% of dentists are women) [39,40]. This demographic shift reflects broader transformations in the structure of medical professions and has important implications for the organization of the labor market and the planning of human resources in healthcare. Although no statistically significant gender differences were identified in the present study, previous research suggests that professional expectations, work–life balance preferences, and career motivations may vary among dental students and healthcare professionals [21,22,23]. Therefore, the observed distribution highlights the importance of considering gender dynamics in future research and in the development of policies aimed at supporting the dental workforce.

### 4.8. Work–Life Balance, and Sustainability of the Profession

The importance assigned by students to work–life balance and supportive work environments should be interpreted in the broader context of professional burnout. Burnout among dental professionals has been widely documented and is associated with high workload, emotional stress, and the demands of clinical practice [20]. The emphasis placed by students on work–life balance may reflect an early awareness of these challenges and a concern regarding long-term professional well-being. Previous studies suggest that career expectations, work environment characteristics, and professional support are important factors influencing job satisfaction and adaptation among dental professionals [5,6,20]. In this context, the need for structured transition support, including mentorship and practical training, may contribute to facilitating professional adaptation during the early stages of practice and improving long-term professional satisfaction and retention.

### 4.9. Implications for Policy and Practice

The findings of this study suggest that improving the transition from dental education to professional practice requires a coordinated approach involving universities, healthcare institutions, and public authorities. Rather than acting solely as employers, public authorities may play a strategic role as facilitators of professional integration through the development of supportive frameworks, partnerships, and targeted interventions. Potential measures include the implementation of structured transition programs, expansion of postgraduate training opportunities, financial and logistical support for establishing practices, and incentives for working in underserved areas. Similar approaches have been implemented in countries such as France and the Netherlands, where they have been associated with improved workforce distribution and professional retention [41]. Taken together, these findings highlight the need for coordinated efforts between educational institutions and public authorities to support the transition of dental graduates into the labor market.

This study has several limitations that should be acknowledged. First, although the sample size (n = 216) exceeded the minimum required number of participants for the target population, participation was voluntary, as the questionnaire was distributed to all eligible students. This may introduce selection bias. In addition, the response rate of approximately 45% may contribute to non-response bias, as students who chose to participate may differ systematically from those who did not respond. These aspects may affect the representativeness of the findings and should be considered when interpreting the results. Second, the study was conducted within a single academic institution (“Carol Davila” University of Medicine and Pharmacy, Bucharest), which may not fully reflect the perspectives of dental students from other universities in Romania. Third, the gender distribution of respondents was not balanced, with a predominance of female participants. This may reflect the actual gender distribution within dental education programs, where female students are often overrepresented, but it may also introduce response bias. Another limitation of this study is related to the use of a non-validated questionnaire. Although the instrument was developed based on a review of relevant literature and underwent a pretesting phase to ensure clarity and face validity, no formal validation procedures (e.g., reliability testing) were performed. Given the exploratory design, inferential analyses were limited to selected associations, which may restrict the generalizability of statistical findings. Finally, as the study is based on self-reported data collected through an online questionnaire, the results may be influenced by subjective perceptions and potential response bias.

## 5. Conclusions

Final-year dental students reported several challenges in the transition from academic training to professional practice, particularly related to limited practical experience and perceived lack of preparedness.

The results indicate a predominant orientation toward private sector employment, while the public sector appears to have reduced visibility among future graduates.

Students expressed a clear need for structured institutional support, including mentorship, postgraduate practical training, career guidance, and entrepreneurship support.

Public authorities may play an important role in facilitating early-career integration through targeted policies, partnerships with educational institutions, and measures aimed at improving workforce distribution.

Overall, improving the transition to professional practice requires actions that include the strengthening of clinical training and practical exposure, development of structured mentorship and transition programs, enhanced collaboration between universities and healthcare systems and increased involvement of public authorities in supporting early-career integration.

## Figures and Tables

**Table 2:** Associations between job selection criteria (Q6) and expected forms of institutional support (Q12).

Q6 Variable	Q12 Variable	χ^2^	*p*-Value
Salary	Career counseling	5.08	0.024
Salary	Entrepreneurship	8.55	0.003
Location	Career counseling	6.26	0.012
Location	Entrepreneurship	4.29	0.038
Private sector	Entrepreneurship	16.0	<0.001
Private sector	Mentoring	5.14	0.023
Public sector	Relocation	7.34	0.007
Job security	Mentoring	4.44	0.035

**Table 3:** Summary of significant associations between preferred professional direction (Q3) and expected forms of institutional support (Q12).

Q3 Variable (Professional Direction)	Q12 Variable (Support Type)	χ^2^	*p*-Value
Privatesector	Entrepreneurship support	16.0	<0.001
Private sector	Mentoring programs	5.14	0.023
Public sector	Relocation support	7.34	0.007

**Table 4:** Summary of significant associations between intention to work abroad (Q8) and expected forms of support (Q12).

Association	χ^2^(df)	*p*-Value	Interpretation
Q8 × Q12 (overall)	14.7 (2)	<0.001	Significant

## Data Availability

The original contributions presented in this study are included in the article and Supplementary Material. Further inquiries can be directed to the corresponding authors.

## Supplementary Materials

The following supporting information can be downloaded at: https://www.mdpi.com/article/10.3390/dj14050300/s1; File S1: Questionnaire for final-year dental students.

## Abbreviations

The following abbreviations are used in this manuscript:

UMFCD	Carol Davila University of Medicine and Pharmacy
