# Supplementary material for: Dental Students’ Perceptions of Workforce Readiness, Career Aspirations and Institutional Support Needs at the Point of Professional Transition: A Cross-Sectional Study in Romania
Source: Dent J (Basel). 2026 May 14;14(5):300. doi: 10.3390/dj14050300 (PMC13205307; doi:10.3390/dj14050300)
Supplement: Supplementary file 1 [file dentistry-14-00300-s001.zip › Questionaaires (english) - Questionnaire The Future in Dentistry What Do You Choose English form.pdf]

The Future in Dentistry – What Do You Choose?

You are invited to participate in a study aimed at evaluating the perceptions of final-year students of the Faculty of Dentistry at the Carol Davila University of Medicine and Pharmacy regarding the transition to professional practice and integration into the labor market.

Participation is voluntary, with no financial or academic rewards. There are no risks associated with participation and completing the questionnaire takes approximately 3–5 minutes.

The questionnaire is anonymous. No personal data (name, email, or any other information that could allow participant identification) are collected. The responses will be used exclusively for scientific purposes and will be analyzed in aggregate form. Participation in the study is voluntary; you may stop completing the questionnaire at any time without consequences.

By continuing to complete the questionnaire, you confirm that:

- you have read the above information
- you are participating voluntarily in the study
- you are a final-year dental student at UMFCD
- you agree to the anonymous use of data for scientific purposes

* Indicates required question

I. General Information

1. Sex: *

Mark only only one.

- ○ Female
- ○ Male
- ○ Prefer not to say

2. Academic year: *

Mark only one.

- ○ Fifth Year
- ○ Sixth Year

II. Career Options

3. ***What is the main professional direction you are aiming for after graduation?*** *

Mark only one.

- Corporate dental practice / network of clinics in Romania – employee/collaborator
- Private practice abroad – employee/collaborator
- Public system (including hospitals, clinics of the national health service)
- Academic career
- Scientific research
- Own private practice or group practice
- Career reorientation (medical representative for a company, etc.)
- I do not know yet

4. ***Which of the following specialties interest you the most?*** * (You may select more than one option)

Tick all that apply.

- Orthodontics and Dentofacial Orthopaedics
- Oral and maxillofacial surgery
- Dento-alveolar surgery
- General dentistry
- Prosthodontics
- Endodontics
- Periodontology
- Pedodontics
- Any of them
- I am not interested in any specialization

III. Practical Training and Integration into the Labor Market

5. Do you feel prepared to enter the labor market after graduation? *

Mark only one.

- Yes

- No
- Partially

6. What are the most important criteria for choosing a job? * (Please select all options that apply to you)

Tick all that apply.

- Salary / Remuneration
- Geographical location
- Private sector
- Public sector
- Professional environment with opportunities for professional development
- Job security
- Clinic reputation
- Flexible schedule
- Friendly work environment
- Respect for and appreciation of the dignity of the profession by the employer
- Opportunity to express commitment toward patients / personal satisfaction
- Opportunity to make use of the knowledge and skills acquired
- Possibility of achieving a balance between professional and personal life
- Other:____

***7. Would you prefer to work in*:** *

Mark only one.

- Urban
- Rural
- Indifferent

8. Do you intend to work abroad? *

Mark only one.

- Yes
- No
- Maybe

9. ***What difficulties do you identify in choosing a workplace after graduation?**** (Please select all options that apply)

Tick all that apply.

- o Lack of opportunities in certain geographical areas
- o High competition in large cities
- o Lack of job positions in the desired area
- o Lack of financial resources
- o Lack of experience
- o Limited opportunities in Romania
- o Lack of support from local/central administration
- o Other:____

10. ***Do you consider that local authorities (city halls, county councils) should be involved in facilitating the professional integration of graduates? ****

Mark only one.

- o Yes
- o No
- o I do not know

11. ***In what way do you think local authorities could support the integration of young dentists into the labor market? **** (Please select all options that apply)

Tick all that apply.

- o Subsidizing the establishment of dental practices in underserved areas
- o Providing low-rent office spaces
- o Increasing the number of positions in the public system
- o Creating partnerships with educational institutions and local practices
- o Information campaigns regarding local opportunities
- o Creating opportunities for family development (land, housing, kindergartens, schools, etc.)
- o Other:____

12. ***What type of support do you consider useful for your professional development? **** (Please select all options that apply)

Tick all that apply.

- Professional mentorship
- Postgraduate internships (traineeships)
- Workshops for developing practical skills
- Career counseling for choosing a specialization
- Guides for entrepreneurship and opening a private practice
- Support for relocation to rural/small urban areas
- Other:____

This content is neither created nor endorsed by Google.

Google Forms
