# Supplementary material for: Dental Students’ Perceptions of Workforce Readiness, Career Aspirations and Institutional Support Needs at the Point of Professional Transition: A Cross-Sectional Study in Romania
Source: Dent J (Basel). 2026 May 14;14(5):300. doi: 10.3390/dj14050300 (PMC13205307; doi:10.3390/dj14050300)
Supplement: Supplementary file 1 [file dentistry-14-00300-s001.zip › Questionaaires (romanian) - Chestionar_STUDENTI - Formulare Google.pdf]

Viitorul în stomatologie – Tu ce alegi?

Sunteți invitat(ă) să participați la un studiu care își propune evaluarea percepției studenților din anii terminali ai Facultății de Medicină Dentară a UMFCD privind tranziția către practica profesională și inserția pe piața muncii.

Participarea este voluntară, fără recompense financiare sau academice. Nu există riscuri asociate participării, iar completarea chestionarului durează aproximativ 3–5 minute.

Chestionarul este anonim. Nu sunt colectate date cu caracter personal (nume, e-mail sau alte informații care ar putea permite identificarea participanților). Răspunsurile vor fi utilizate exclusiv în scop științific și vor fi analizate în formă agregată. Participarea la studiu este voluntară, puteți întrerupe completarea chestionarului în orice moment, fără consecințe.

Prin continuarea completării chestionarului, confirmați că:

- ați citit informațiile de mai sus
- participați voluntar la studiu
- sunteți student în an terminal la Medicină Dentară - UMFCD
- sunteți de acord cu utilizarea anonimă a datelor în scop științific

* Indică o întrebare obligatorie

Treci la întrebarea 1Treci la întrebarea 1

I. Date generale

1. 1. Sex: *

Marchează un singur oval.

- Feminin
- Masculin
- Prefer să nu spun

2. 2. Anul de studiu: * Dropdown

Marchează un singur oval.

- anul V
- anul VI

II. Opțiuni de carieră

3. 3. Care este direcția profesională principală pe care o vizezi după absolvire? *

Marchează un singur oval.

- Cabinet stomatologic corporativ/rețea de clinici în România – angajat/colaborator
- Cabinet privat în străinătate – angajat/colaborator
- Sistem public (inclusiv spitale, clinicile serviciului national de sănătate)
- Carieră academică
- Cercetare științifică
- Practică privată proprie sau de grup
- Reorientare în carieră (reprezentant medical al unei companii etc)
- Nu știu încă

4. 4. Care dintre specializări te interesează mai mult? (Puteți selecta mai multe variante) *

Bifează toate variantele aplicabile.

- [ ] Ortodonție și ortopedie dento-facială
- [ ] Chirurgie stomatologică și maxilo-facială
- [ ] Chirurgie dento-alveolară
- [ ] Stomatologie generală
- [ ] Protetică dentară
- [ ] Endodonție
- [ ] Parodontologie
- [ ] Pedodonție
- [ ] Oricare
- [ ] Nu mă interesează nici o specializare

III. Pregătirea practică și inserția pe piața muncii

5. 5. Te simți pregătit(ă) să intri pe piața muncii după absolvire? *

Marchează un singur oval.

- Da
- Nu
- Parțial

6. 6. Care sunt cele mai importante criterii pentru alegerea unui loc de muncă? (Vă rugăm să selectați toate variantele care vi se potrivesc) *

Bifează toate variantele aplicabile.

- [ ] Salariul/Onorariul
- [ ] Locația geografică
- [ ] Sistemul privat
- [ ] Sistemul public
- [ ] Mediu profesional cu oportunități de dezvoltare profesională
- [ ] Siguranța locului de muncă
- [ ] Reputația clinicii
- [ ] Program flexibil
- [ ] Colectiv prietenos
- [ ] Respectul și aprecierea demnității profesiei de către angajator
- [ ] Posibilitatea de a exprima atașamentul față de pacienți/satisfacția personală
- [ ] Oportunitatea de a valorifica cunoștiințele și deprinderile acumulate
- [ ] Posibilitatea creării echilibrului între viața profesională și personală
- [ ] Altele: ____________________

7. 7. Ai prefera să lucrezi în mediul: *

Marchează un singur oval.

- ( ) Urban
- ( ) Rural
- ( ) Indiferent

8. 8. Intenționezi să lucrezi în străinătate? *

Marchează un singur oval.

- ( ) Da
- ( ) Nu
- ( ) Poate

9. 9. Ce dificultăți identifici în alegerea unui loc de muncă după finalizarea studiilor? (Puteți selecta mai multe variante) *

Bifează toate variantele aplicabile.

- [ ] Lipsa oportunităților în anumite zone geografice
- [ ] Concurență mare în orașele mari
- [ ] Lipsa locurilor de muncă în zona dorită
- [ ] Lipsa resurselor financiare
- [ ] Lipsa experienței
- [ ] Oportunități limitate în România
- [ ] Lipsa suportului administrației locale/centrale
- [ ] Altele: ____________________

10. 10. Consideri că autoritățile locale (primării, consilii județene) ar trebui să se implice în facilitarea inserției profesionale a absolvenților? *

Marchează un singur oval.

- ( ) Da
- ( ) Nu
- ( ) Nu știu

11. 11. În ce mod crezi că autoritățile locale ar putea sprijini integrarea tinerilor medici stomatologi pe piața muncii? (Puteți selecta mai multe variante) *

Bifează toate variantele aplicabile.

- [ ] Subvenționarea deschiderii de cabinete în zone defavorizate
- [ ] Oferirea de spații închiriabile la preț redus
- [ ] Creșterea numărului de posturi în sistemul public
- [ ] Crearea unor parteneriate cu instituții de învățământ și cabinete locale
- [ ] Campanii de informare privind oportunitățile locale
- [ ] Crearea oportunităților de dezvoltare familială (teren, locuință, grădinițe, școli etc)
- [ ] Altele: ____________________

12. 12. Ce tip de sprijin consideri că ar fi util pentru dezvoltarea ta profesională? (Puteți selecta mai multe variante) *

Bifează toate variantele aplicabile.

- [ ] Mentorate profesionale
- [ ] Stagii de practică postuniversitară (stagiaturi)
- [ ] Workshopuri pentru dezvoltarea abilităților practice
- [ ] Consiliere pentru alegerea unei specializări
- [ ] Ghiduri pentru antreprenoriat și deschiderea unui cabinet propriu
- [ ] Suport pentru relocare în mediul rural/urbanul mic
- [ ] Altele: ____________________

Acest conținut nu este nici creat, nici aprobat de Google.

Formulare Google
